# Lymphadenectomy in bladder cancer: What should be the extent?

**DOI:** 10.4103/0970-1591.70593

**Published:** 2010

**Authors:** K Muruganandham, A Mandhani

**Affiliations:** Department of Urology and Renal Transplantation, Sanjay Gandhi Post Graduate Institute of Medical Sciences, Lucknow, UP, India

**Keywords:** Bladder cancer, lymphadenectomy, radical cystectomy

## Abstract

The extent of Lymh node dissection (LND) during radical cystectomy is a subject of increasing importance with several studies suggesting that an extended LND may improve staging accuracy and outcome. Significant numbers of patients have lymph node metastasis above the boundaries of standard LND. Extended LND yields higher number of lymph nodes which may result in better staging. Various retrospective studies have reported better oncological outcomes with extended LND compared to limited LND. No difference in the mortality and the incidence of lymphocele formation has been found between ‘standard’ and ‘extended’ LND. Till we have a well-designed randomized controlled trial to address these issues for level 1 evidence, it is not justified to deny our patients the advantages of ‘extended’ lymphadenectomy based on the current level of evidence.

## INTRODUCTION

Radical cystectomy (RC) with bilateral pelvic lymph node dissection (LND) is the standard treatment for invasive bladder cancer done with the intention to enhance local disease control and possibly to improve overall survival. However, the extent of LND has not been standardized and its relevance for staging and prognosis is still not clear. Boundaries of ‘standard’ LND include bifurcation of common iliac artery superiorly, the genitofemoral nerve laterally, the circumflex iliac vein and lymph node of Cloquet distally, and the hypogastric vessels posteriorly. In ‘extended’ LND, lateral and distal limits are similar to the ‘standard’ LND and it involves removal of presacral nodes also. The superior extent is not well defined and has been described as the aortic bifurcation,[1] 1–2 cm above the aortic bifurcation[2] or up to the inferior mesenteric artery[3] by various authors. The extent of LND is a subject of increasing importance with several studies suggesting that an extended LND may improve staging accuracy and outcome.

## EVIDENCE

### 

#### Pattern of lymph node metastasis in bladder cancer

A prospective lymph node mapping study showed that though the positive lymph nodes were most common in the obturator and the iliac groups, 16% of lymph node metastases were above the aortic bifurcation while 8% of nodal metastases were in the presacral region. Among patients with nodal metastases located within the limits of a ‘standard’ dissection, a significant proportion also had nodal involvement at the level of the common iliac vessels and above the aortic bifurcation (57 and 31%, respectively). Furthermore, there were no isolated nodal metastases above the aortic bifurcation, suggesting that ‘skip lesions’ are rare.[3] Vazina *et al*. reported that 24.4% had lymph nodes involvement at places other than the ‘standard’ template and 9% had disease above the common iliac bifurcation while ‘skip metastasis’ above the common iliac bifurcation was reported in only one patient, which was also attributed to labeling error.[4] In another study Steven *et al*. reported that 34% of patients had positive nodes above the common iliac bifurcation.[2]

#### Whether ‘extended’ lymph node dissection yields higher nodes and improves staging?

By extending the boundaries of the LND, Poulsen *et al*. reported an increase in the mean number of lymph nodes removed (25 vs. 14 in a standard dissection) and Bochner *et al*. confirmed these findings, reporting significantly more nodes removed with an ‘extended’ LND (36.5 vs. 8.5 nodes).[5,6] In a retrospective comparative study between two high-volume centers higher number of lymph nodes was removed with ‘extended’ LND compared to ‘standard’ LND (22 vs. 12 nodes).[1] All these studies demonstrate that extended LND yields higher number of lymph nodes which may result in better staging.

#### Whether ‘extended’ lymph node dissection improves outcome?

Radical cystectomy including ‘extended’ LND was reported to improve survival by 21% compared to that obtained by ‘standard’ LND for patients with invasive bladder cancer confined to the bladder wall.[5] In another study, RC with ‘extended’ LND with the removal of more than 16 lymph nodes resulted in a 22% five-year survival advantage.[7] Steven *et al*. reported that in 336 patients who underwent RC with ‘extended’ LND the overall five-year survival rates was similar in patients with lymph node involvement above the bifurcation of the common iliac vessels and in those with lymphatic metastases confined to the true pelvis.[2] In a recent study, Dhar *et al*. retrospectively compared outcomes in patients undergoing RC who either had ‘standard’ (patients at Cleveland clinic, *N* = 336) and ‘extended’ LND (patients at Bern University, *N*=322). The overall lymph node positive rate and five-year survival was 13% and 7% for patients with standard and 26% and 35% for those who had extended pelvic lymph node dissection, respectively.[1] Polymerase chain reaction analysis revealed bladder cancer associated DNA in the lymph nodes of 29% to 33% of patients classified as having node negative disease by standard histopathology and the survival advantage with extended LND may result from a more complete excision of these lymph nodes harboring micrometastasis.[8]

#### Whether ‘extended’ lymph node dissection leads to excess morbidity?

In the study by Brossner *et al*. 46 patients undergoing an extended LND were compared to 46 well-matched patients undergoing ‘standard’ PLND. Although the extended lymphadenectomy increased the operative duration by 63 minutes, there was no significant difference in perioperative mortality, early complications, or the need for blood transfusions between the groups.[9] In another retrospective analysis, there was no difference in the mortality, and the incidence of lymphocele formation between ‘standard’ and ‘extended’ LND.[5]

## CONCLUSION

Although the absolute limits of the lymph node dissection remain to be determined, available evidence supports doing an ‘extended’ LND with RC as it improves staging accuracy and outcome without increasing the morbidity. Despite variation in the extent of superior boundary, dissection up to the aortic bifurcation is a uniform extent in all the studies. Till we have a well-designed randomized controlled trial to address these issues for level 1 evidence, it is not justified to deny our patients the advantages of ‘extended’ lymphadenectomy based on the current level of evidence.
